# Adenylate cyclase‐activating polypeptide 1 gene methylation predicts prognosis and the immune microenvironment of bladder cancer

**DOI:** 10.1002/ctm2.597

**Published:** 2021-10-14

**Authors:** Wenhao Xu, Aihetaimujiang Anwaier, Wangrui Liu, Xi Tian, Yuanyuan Qu, Jianyuan Zhao, Hailiang Zhang, Dingwei Ye

**Affiliations:** ^1^ Department of Urology Fudan University Shanghai Cancer Center Shanghai 200032 P.R. China; ^2^ State Key Laboratory of Genetic Engineering School of Life Sciences, Fudan University Shanghai 200438 P.R. China

Dear editor,

Due to increasing mortality and limited diagnostic methods of bladder cancer (BCa),^1^ early detection and response prediction for BCa signatures are essential for improving prognosis and treatment strategies; however, effective diagnostic methods are limited. Large‐scale high‐throughput sequencing has the potential to greatly improve BCa diagnostics.^2^, ^3^

Approval of immune checkpoint therapies (ICTs) for advanced BCa was a paradigm shift in BCa treatment because ICTs produce durable responses and improve prognosis.^4^ However, internal tumour heterogeneity and multi‐omics changes have limited their success despite robust anti‐tumour responses and multiple ICTs being approved for clinical use.^5^ Previous studies have reported abnormal promoter methylation in BCa but have not fully elucidated the specificity of DNA methylation changes.^6^ Previous studies have reported abnormal promoter methylation in BCa but have not fully elucidated the specificity of DNA methylation changes.^7^ Here, we identified diagnostic methylation markers through differential expression analysis. We then compared the association between methylation of the hub methylation gene *ADCYAP1* and the tumour immune parameters of the microenvironment in various cancer types to validate the promoter region and the accuracy of prognostic BCa markers. The study process is depicted in Figure S1.

First, we screened and identified the methylation‐regulated differentially expressed genes for BCa (Figure S2). Survival analysis suggested that elevated expression of *ADCY2* (*p *= .006), *APP* (*p *= .0015), *BDKRB2* (*p *= .0016), *FPR1* (*p *= .002), *GNB4* (*p *= .0043), *GNG11* (*p *= .0054), *ADCY9* (*p *= .019) and *ADCYAP1* (*p *= 3e‐04) significantly predicted outcomes for BCa patients (Figure S3).

At the same time, we investigated the differential expression and methylation levels of ADCY2 and ADCYAP1 in 12 452 pan‐cancer samples (Figure 1A–D). Besides BCa and adjacent normal tissues, we found significantly differential expression and methylation levels of *ADCY2* and *ADCYAP1* in many others cancers, especially in BCa samples (Figure S4). *ADCYAP1* methylation showed a stronger association with mRNA expression (Cor = –.26, false discovery rate (FDR) = 9.2e‐08) than *ADCY2* (Cor = –.11, FDR = 3.0e‐02; Figure 1E,F). Additionally, sensitive drugs were predicted in Figure 1G, bleomycin and docetaxel have relatively markedly sensitivity to both *ADCY2* and *ADCYAP1*, and PLX4730 showed a significant negative association with *ADCY* mRNA expression. To improve, we hypothesised that the sensitivity of docetaxel treatment may be consistent with *ADCYAP1* mRNA expression in BCa. The findings revealed that *ADCYAP1* overexpression group significantly reduced the adenosine triphosphate synthase activity under different concentrations of docetaxel (1, 2, 3, 5, 8, 12 nmol/L) in cultured human urinary bladder carcinoma 5637 and T24 cells (Figure S5). Importantly, to explore the role of *ADCY2* and *ADCYAP1* in intratumoural heterogeneity, we evaluated the correlation between expression levels of hub genes and tumour microenvironment. It suggested that *ADCYAP1* showed remarkable association with infiltration score, an abundance of CD^8+^ T cells, cytotoxic T cells, macrophage, neutrophil and natural killer (NK) cells infiltration (Figure 1H,I).

**FIGURE 1:**
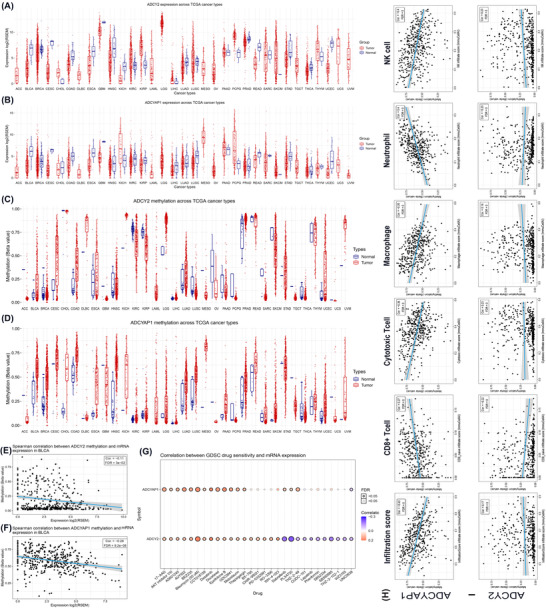
Differential expression, methylation and immune correlation of hub genes *ADCY2* and *ADCYAP1* in pan‐cancers from the Cancer Genome Atlas (TCGA). (A–D) Differential expression and methylation levels of *ADCY2* and *ADCYAP1* in 12 452 pan‐cancer samples. (E–F) Associations between methylation and mRNA levels of *ADCYAP1* and *ADCY2* (Spearman correlation test). (G) Sensitivity of bladder cancer (BCa) to drugs was predicted with respect to methylation levels of *ADCY2* and *ADCYAP1* based on the top 30 Genomics of Drug Sensitivity in Cancer (GDSC) drugs in pan‐cancer. (H) Correlations between the levels of methylation in *ADCY2* and *ADCYAP1* and immune parameters

To further explore the predictive role of *ADCYAP1* in tumour immune microenvironment and responses to ICTs of BCa, we divided 408 BCa patients into *ADCYAP1* ^high^ and *ADCYAP1* ^high^ groups based on expression levels. To obtain reliable immune infiltration estimations, we found that high expression of *ADCYAP1* significantly correlated with decreased T cells, endothelial cells, NK cells, mast cells and elevated macrophages infiltration, specifically M2 macrophages (Figure 2A–C). Next, we found that *ADCYAP1* ^high^ significantly correlated with increased expression of *HAVCR2*, *PDCD1LG1*, *LAG3*, *CTLA4*, *PDCD1* and *TIGIT* (Figure 2D,E). In 12 452 tumour samples, *ADCYAP1* expression exhibited a markedly positive relationship with microenvironment score, immune score, monocyte, mast cell, endothelial cell, myeloid progenitor and CD^8+^ T cells, shaping pro‐tumourigenic immune infiltration of cancers (Figures 2F and S6).

**FIGURE 2:**
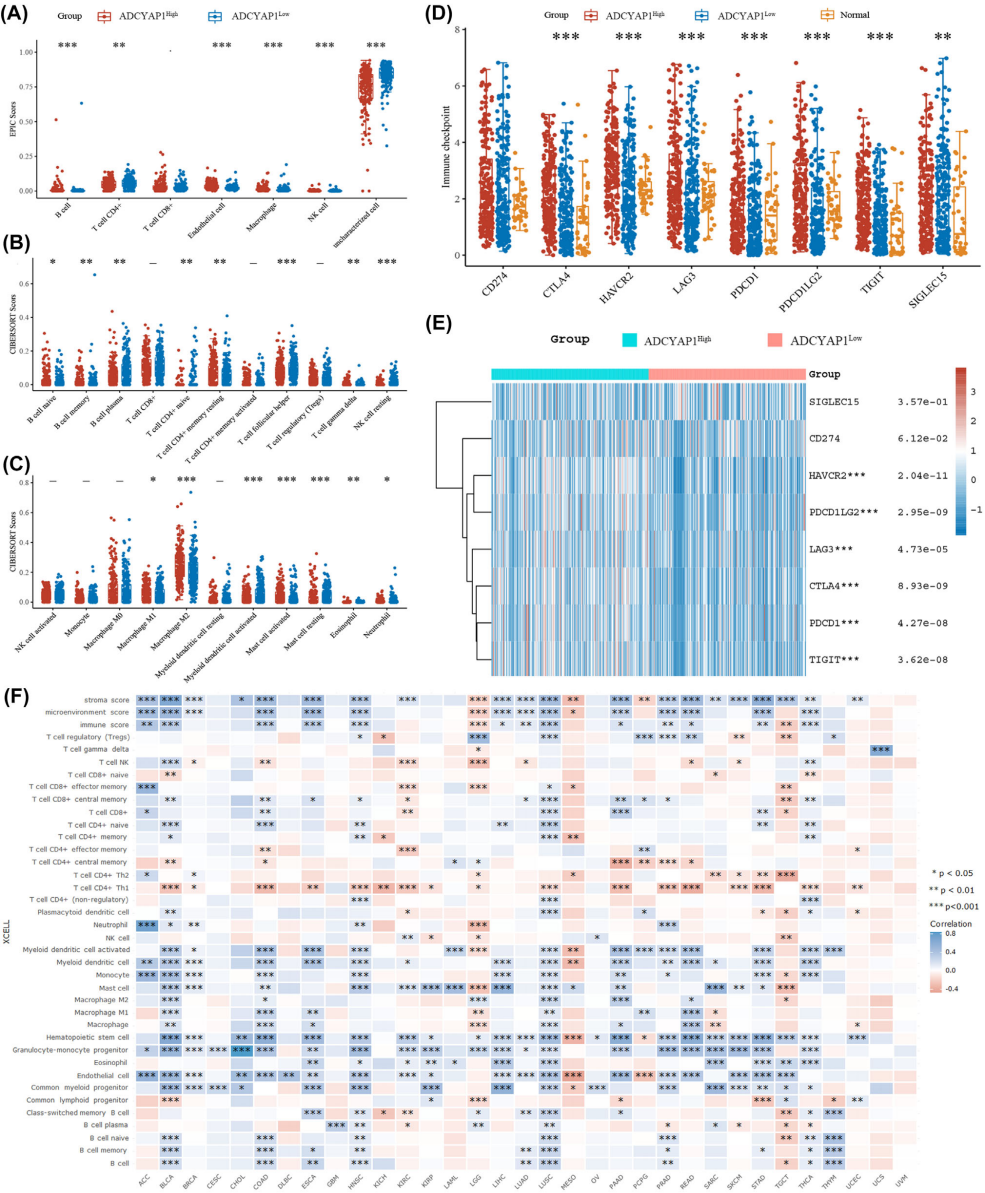
Predictive role of *ADCYAP1* expression for the immune microenvironment in BCa and pan‐cancers. (A–C) To further explore the predictive role of *ADCYAP1* expression with respect to BCa tumour immune parameters and responses to immune checkpoint therapies (ICTs), we divided 408 BCa patients into *ADCYAP1* ^high^ and *ADCYAP1* ^low^ groups. To obtain reliable immune infiltration estimations, we utilised ‘immunedeconv’, an R package program that integrates CIBERSORT and EPIC algorithms. (D–E) Differential expression of immune checkpoint molecules in normal samples, *ADCYAP1* ^low^ and *ADCYAP1* ^high^ tumour samples (Kruskal–Wallis test). (F) In 12 452 tumour samples, *ADCYAP1* expression exhibited markedly positive relationships with immune parameters, including immune score, monocytes, mast cells, endothelial cells, myeloid progenitors and CD^8+^ T cells, shaping pro‐tumourigenic immune infiltration of cancers

However, the expression of *ADCYAP1* and the potential regulatory mechanisms in BCa have not been fully elucidated and are worth further investigating. Next, we assessed methylation of *ADCYAP1* in BCa cells and selected UMUC3 and T24 cells for further analysis (Figure 3A). CpG islands situated in the promoter region of *ADCYAP1* and the designed bisulfite amplicon sequencing (BSAS) primers are shown in Figure 3B. The methylation sequencing showed that in the comparison between the two cell lines, except for the 186 loci, the methylation levels of the remaining sites were significantly different (Figure 3C,D). We also implemented BSAS to validate the effectiveness of methylation‐specific Polymerase chain reaction (PCR) primers and to evaluate the methylation density of a prolonged genomic sequence in the *ADCYAP1* promoter region. The BSAS analysis proved methylation and unmethylation of *ADCYAP1* in UMUC3 and T24 cells, respectively (Figure 3E).

**FIGURE 3:**
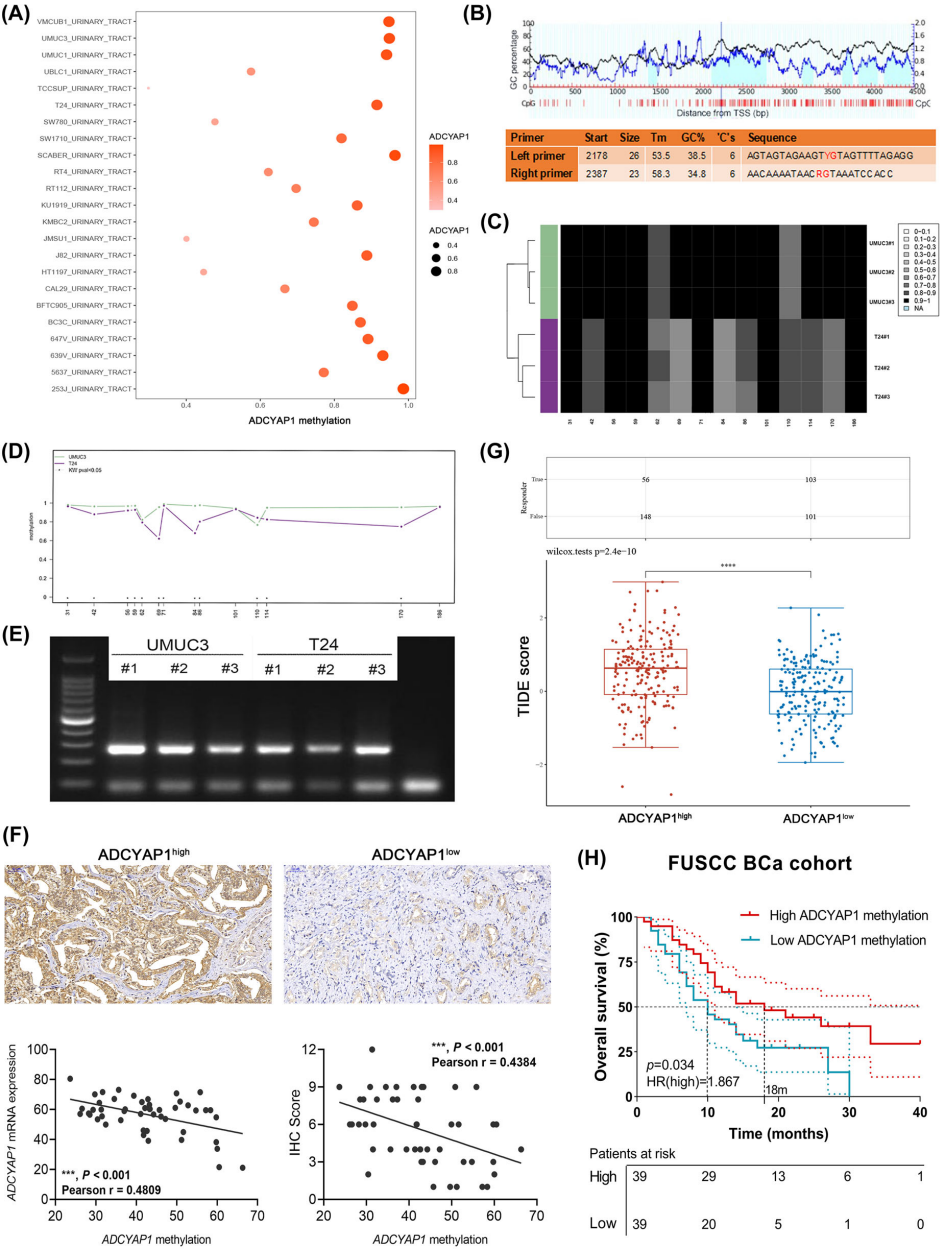
Expression of *ADCYAP1* is regulated by promoter region methylation and can predict immune parameters and responses to immunotherapy in BCa. (A) We assessed the level of *ADCYAP1* methylation in BCa cells and selected UMUC3 and T24 cells for further analysis on the basis of Cancer Cell Line Encyclopedia. (B) CpG islands in the *ADCYAP1* promoter region and the primers designed for bisulfite amplicon sequencing (BSAS). (C–D) Comparison of methylation between the two cell lines. (E) Efficiency of methylation‐specific PCR primers and assessment of methylation density of a prolonged genomic sequence in the *ADCYAP1* promoter region using BSAS. (F) To demonstrate that *ADCYAP1* is regulated by promoter methylation, *ADCYAP1* protein, transcription and methylation expression were evaluated in 49 BCa samples from the Fudan University Shanghai Cancer Center (FUSCC) tissue bank using immunohistochemistry, real‐time quantitative PCR (RT‐qPCR)and methylation‐specific PCR. Pearson's correlation analysis was used to predict the association between *ADCYAP1* methylation and expression levels. (G) Differential TIDE scores were evaluated in high and low *ADCYAP1* expression BCa patient groups using the unpaired *t‐*test. (H) Prognostic value of *ADCYAP1* methylation in 78 BCa patients from the FUSCC cohort receiving ICTs and for whom electronic clinical records and follow‐up data were available

To demonstrate that *ADCYAP1* is regulated by promoter methylation, we first enrolled 49 BCa samples from Fudan University Shanghai Cancer Center tissue bank and evaluated proteomic, mRNA and methylation levels of ADCYAP1 using immunohistochemistry, real‐time quantitative PCR (RT‐qPCR) and PCR, respectively (Figure 3F). It reveals that increased *ADCYAP1* methylation is significantly correlated with decreased mRNA levels (*p *< .001, Pearson's *r* = –.4809) and protein abundance (*p *< .001, Pearson's *r* = –.4384). Interestingly, high *ADCYAP1* expression significantly correlated with elevated Tumor Immune Dysfunction and Exclusion (TIDE) score, suggesting highly heterogeneous tumour microenvironment and poor response to ICTs of BCa (*p *< .0001; Figure 3G). Subsequently, we enrolled 78 BCa patients receiving ICTs in our institute with available electronic clinical records and follow‐up data. After assessing *ADCYAP1* methylation level of BCa tissues, we found that decreased *ADCYAP1* methylation significantly predicted worse overall survival (*p *= .034, HR = 1.867; Figure 3H). Taken together, these results revealed that the *ADCYAP1* expression is affected by the methylation of promoter region, which could significantly predict immune‐infiltrated microenvironment and responses to immunotherapy in BCa.

In conclusion, this study described differential expression and methylation profiles, which improve the prognostic accuracy of biomarkers in BCa. *ADCYAP1* methylation has extensive anti‐tumourigenic immune infiltration of cancers and significantly predict immune‐infiltrated microenvironment and better survival benefits for patients with BCa. Our discovery of the novel independent prognostic indicators in BCa highlights the relationship among tumour phenotype, epigenetics and immune contexture.

## CONFLICT OF INTEREST

The authors declare that there is no conflict of interest.

## FUNDING INFORMATION

This work is supported by Grants from the National Key Research and Development Program of China (No. 2019YFC1316000), Natural Science Foundation of Shanghai (No. 20ZR1413100), “Fuqing Scholar” Student Scientific Research Program of Shanghai Medical College, Fudan University (No. FQXZ202112B), and Shanghai Municipal Health Bureau (No. 2020CXJQ03).

## Supporting information

Supporting Information

Supporting Information

Supporting Information

Supporting Information

Supporting Information

Supporting Information
