# Supplementary figures and images for: Adenylate cyclase‐activating polypeptide 1 gene methylation predicts prognosis and the immune microenvironment of bladder cancer
Source: Clin Transl Med. 2021 Oct 14;11(10):e597. doi: 10.1002/ctm2.597 (PMC8516337; doi:10.1002/ctm2.597)

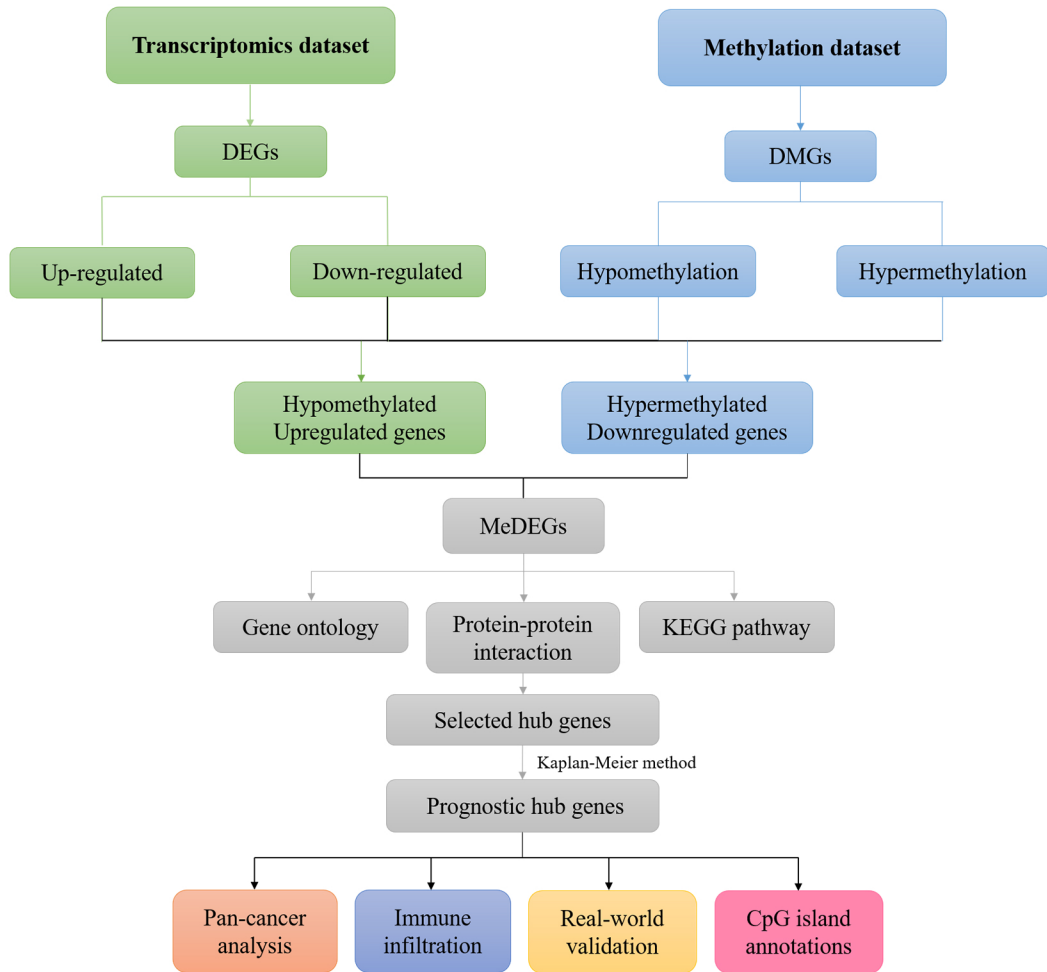

Supplement: Supplementary file 1 — Supporting Information [file CTM2-11-e597-s001.pdf]

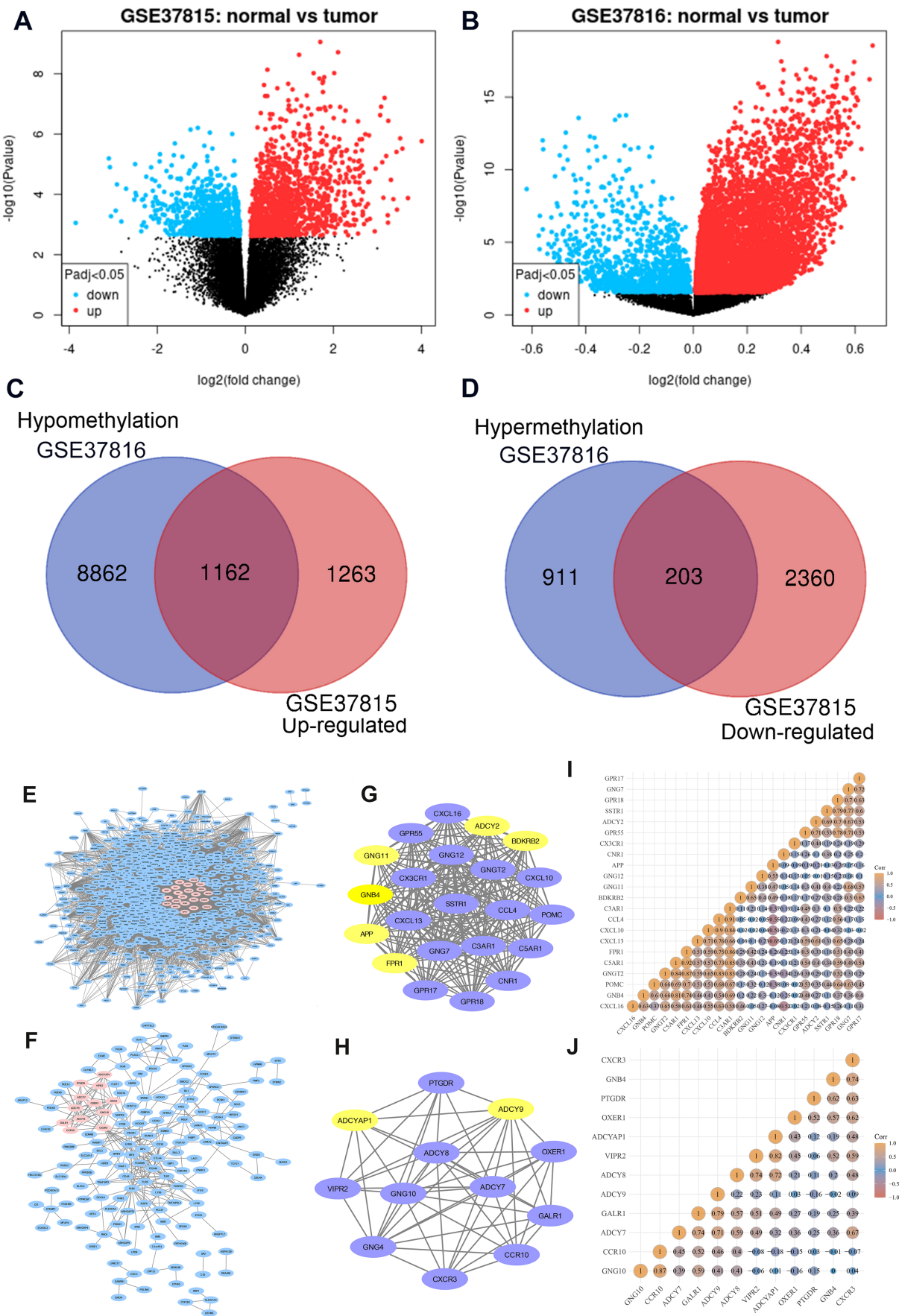

Supplement: Supplementary file 2 — Supporting Information [file CTM2-11-e597-s002.pdf]

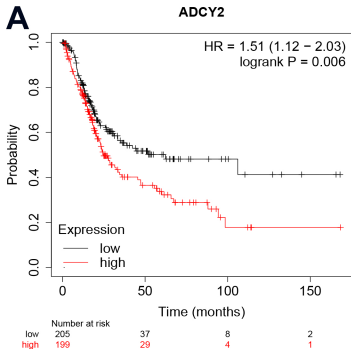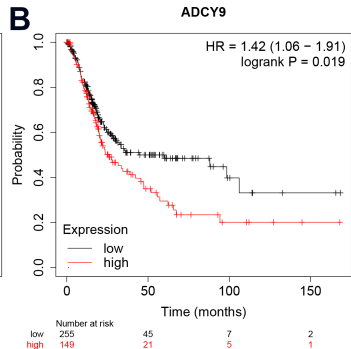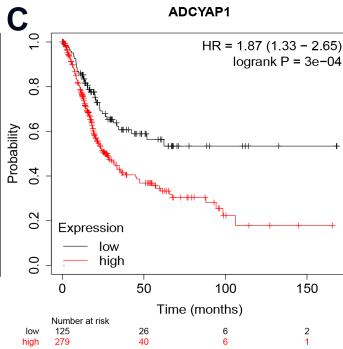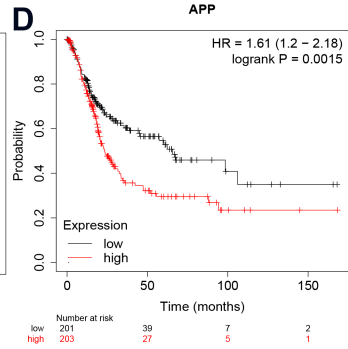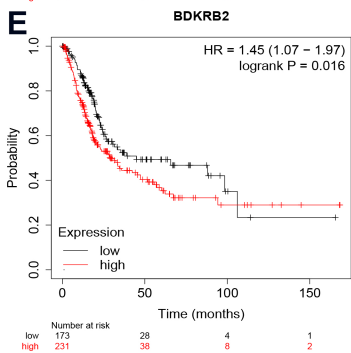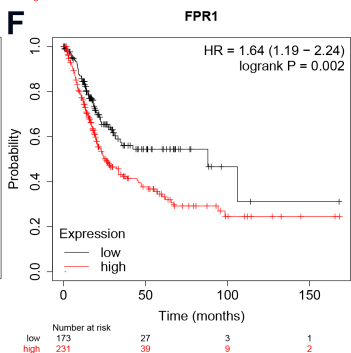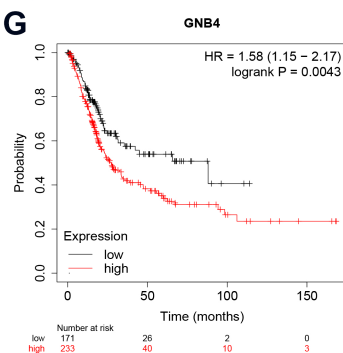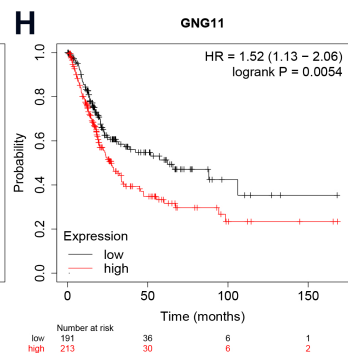

Supplement: Supplementary file 3 — Supporting Information [file CTM2-11-e597-s004.pdf]

# ADCYAP1 methylation in BLCA

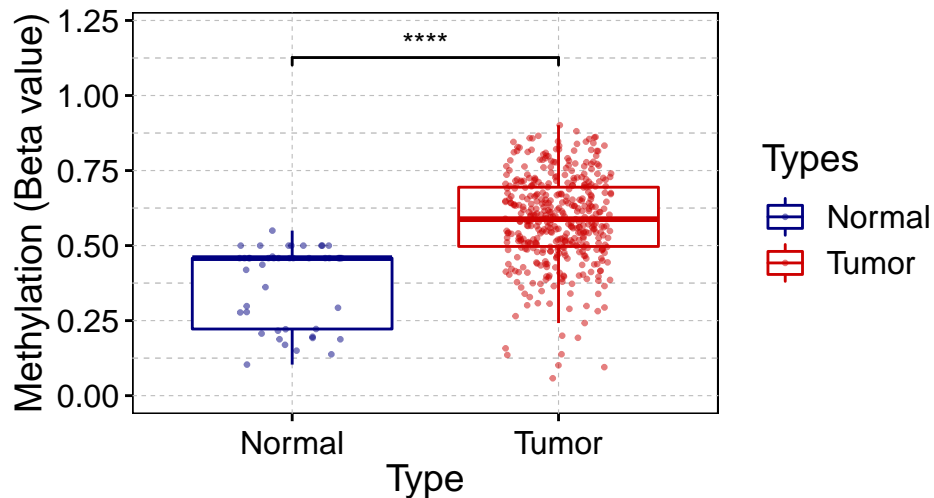

Supplement: Supplementary file 4 — Supporting Information [file CTM2-11-e597-s003.pdf]

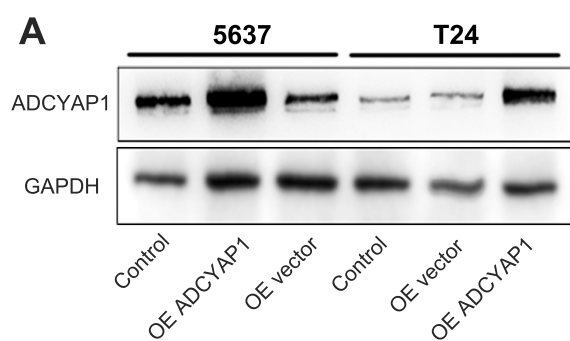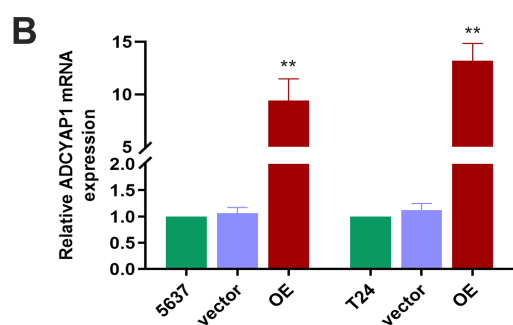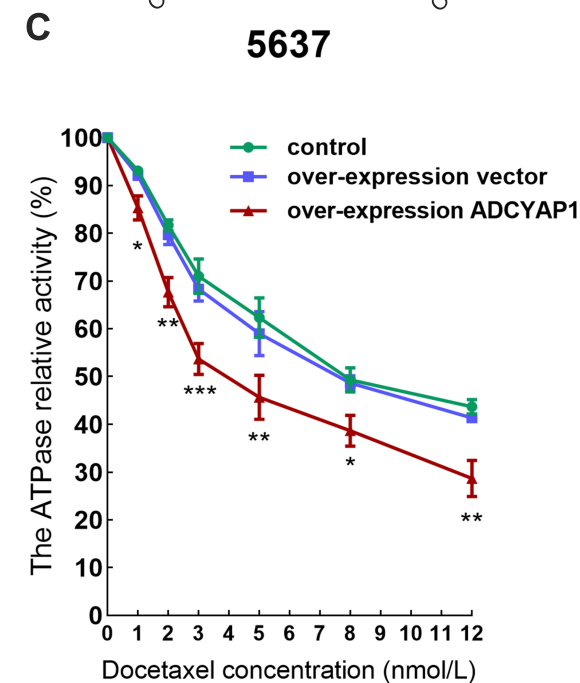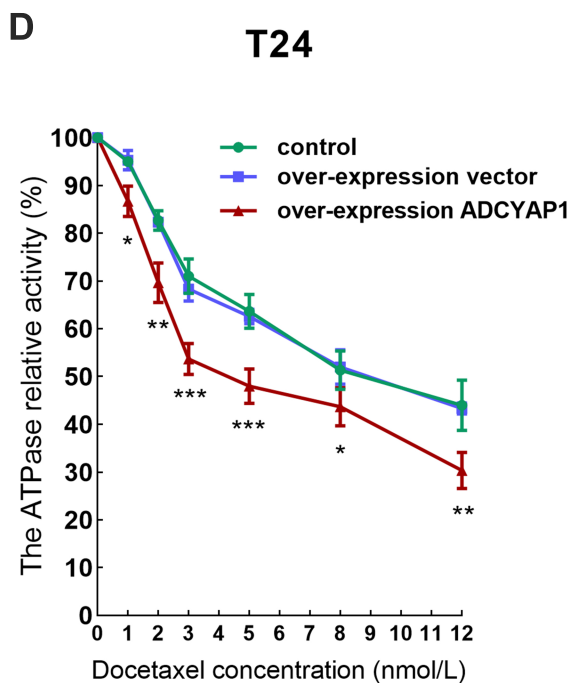

Supplement: Supplementary file 5 — Supporting Information [file CTM2-11-e597-s006.pdf]

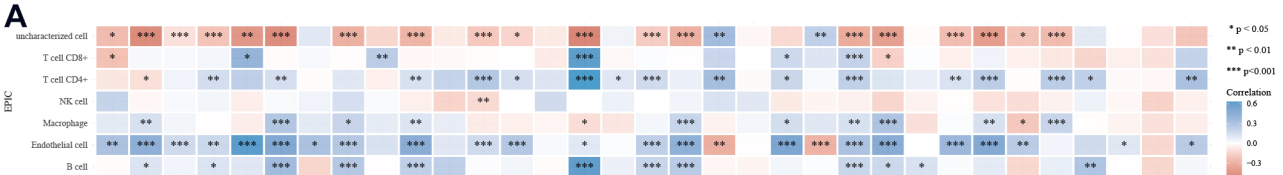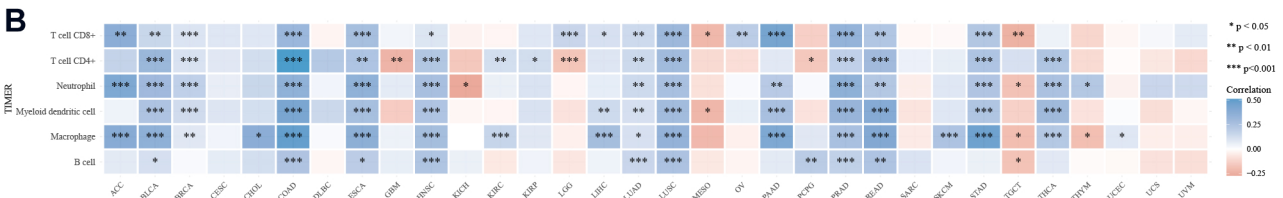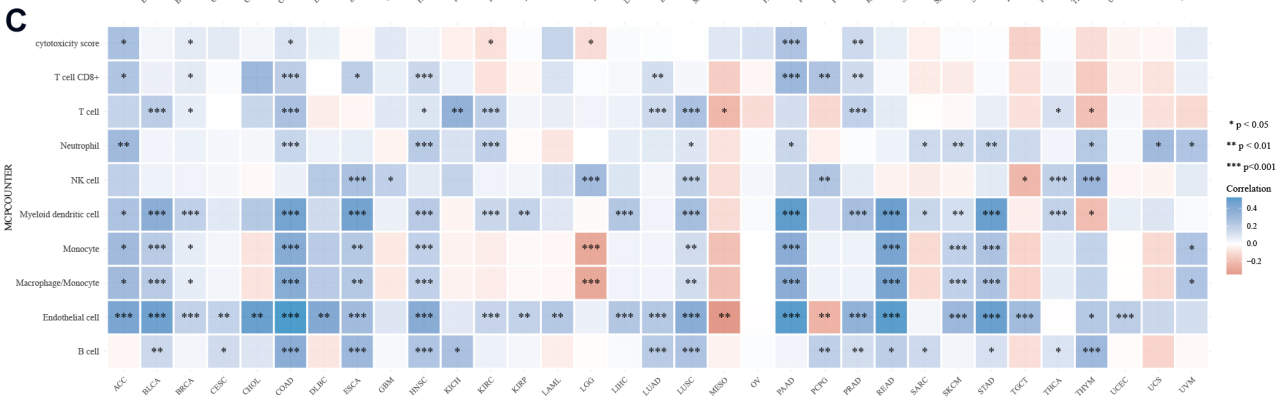

Supplement: Supplementary file 6 — Supporting Information [file CTM2-11-e597-s005.pdf]
